# Efficacy, safety, and pharmacokinetics of teduglutide in adult Japanese patients with short bowel syndrome and intestinal failure: two phase III studies with an extension

**DOI:** 10.1007/s00595-022-02587-4

**Published:** 2022-10-06

**Authors:** Shiro Nakamura, Motoshi Wada, Tsunekazu Mizushima, Akira Sugita, Yuko Tazuke, Hiroki Ohge, Eri Udagawa, Ryohsuke Ken Suzuki, MinJung Yoon, Andrew Grimm, Szu-Ta Chen, Hiroki Ikeuchi

**Affiliations:** 1 Osaka Medical and Pharmaceutical University, Osaka, Japan; 2 Tohoku University Hospital, Sendai, Miyagi Japan; 3 Osaka University Hospital, Suita, Osaka Japan; 4 Yokohama Municipal Citizen’s Hospital, Yokohama, Kanagawa Japan; 5 Hiroshima University Hospital, Hiroshima, Japan; 6 Takeda Pharmaceutical Company Limited, Tokyo, Japan; 7 Takeda Pharmaceutical Company, Cambridge, MA USA; 8 Shire Human Genetic Therapies, Inc., a Takeda Company, Cambridge, MA USA; 9 Hospital of Hyogo College of Medicine, Nishinomiya, Hyogo Japan; 10 Present Address: Ultragenyx Pharmaceutical Inc, Novato, CA USA

**Keywords:** Parenteral support (PS), Short bowel syndrome (SBS), Intestinal failure (IF), Teduglutide

## Abstract

**Purpose:**

The short- and long-term efficacy, safety, and pharmacokinetics of teduglutide were analyzed in adult Japanese patients with short bowel syndrome and intestinal failure (SBS-IF).

**Methods:**

Patients received teduglutide 0.05 mg/kg/day in clinical trials (TED-C14-004, SHP633-306, and extension SHP633-307). Data were analyzed at 24 weeks and an interim data cut-off of 4.5 years.

**Results:**

The parenteral support (PS) volume decreased by ≥ 20% for 9/18 patients at 24 weeks and in all 11 patients by data cut-off in SHP633-307. The mean (standard deviation) PS volume decreased from baseline at 24 weeks in TED-C14-004 (−30.1 ± 25.9%) and SHP633-306 (−25.6 ± 25.5%), and at data cut-off in SHP633-307 (−57.08 ± 28.49%). Teduglutide was absorbed quickly. The adverse events were consistent with the underlying disease and known adverse drug reactions. Anti-teduglutide antibody titers declined with long-term treatment.

**Conclusions:**

In Japanese adults with SBS-IF, teduglutide treatment was associated with clinically meaningful reductions in PS requirements, similar to findings in prior international studies. No new safety concerns specific to the Japanese SBS-IF patient population were identified with short- or long-term teduglutide treatment. Anti-teduglutide antibody titers disappeared in most Japanese adults with long-term treatment. These results constitute the longest evaluation of teduglutide treatment within clinical trials reported to date.

**Supplementary Information:**

The online version contains supplementary material available at 10.1007/s00595-022-02587-4.

## Introduction

Short bowel syndrome (SBS) is a rare condition resulting from significant physical or functional loss of portions of the bowel and is the most common cause of intestinal failure (IF) [1, 2]. IF associated with SBS (SBS-IF) leads to a reduction in the gut function below the minimum level required to maintain absorption of macronutrients, water, and electrolytes in order to sustain health and/or growth [3, 4].

Patients with SBS-IF comprise a heterogeneous population differing in underlying pathology, the function of the remnant bowel, and demographic characteristics [5]. Demographic differences between Japanese and Western populations, including a reduced prevalence of being overweight and obese in Japanese patients, combined with differences in nutritional management of SBS, may result in an increased risk of undernutrition among Japanese patients with SBS [6, 7].

SBS in adults is commonly caused by extensive intestinal resection for recurrent Crohn’s disease, mesenteric ischemia, intestinal obstruction, or malignancy, and may involve partial or complete colonic resection [5]. After resection, the intestine may expand its mucosal surface area to increase the absorptive capacity [4]. This adaptive response is driven in part by release of glucagon-like peptide 2 (GLP-2) from enteroendocrine L cells in the distal ileum and proximal colon [8, 9]. However, extensive surgical resection of these regions can impair this adaptive capacity.

Severe reductions in the intestinal absorptive capacity may render patients with chronic SBS-IF dependent on life-sustaining parenteral support (PS; parenteral nutrition and/or intravenous fluids) [4]. A 12-year observational study focused on patients in Japan with SBS-IF found that most patients could not be weaned off PS and that older adults had lower weaning rates than younger adults [10]. Long-term administration of PS is associated with complications that are potentially life-threatening, including IF-associated liver disease (IFALD), sepsis central line-associated bloodstream infections, and central venous thrombosis [10, 11].

Teduglutide is a GLP-2 analogue approved in several countries and continents, including the United States and Europe (2012), for the treatment of patients ≥ 1 year old with SBS-IF (0.05 mg/kg subcutaneously [SC] once daily) who are dependent on PS [12, 13]. It has also recently been approved in Japan (2021) for the treatment of patients with a corrected age of ≥ 4 months and a body weight of ≥ 10 kg [14, 15]. Teduglutide markedly reduced the need for PS in adults with SBS-IF in two randomized international (patients recruited from the United States, Canada, and Europe), placebo-controlled, pivotal phase III studies (CL0600-004 and STEPS-1 [Study of Teduglutide Effectiveness in Parenteral Nutrition-Dependent Short Bowel Syndrome Subjects], completed in 2010 and 2011) [16, 17] and their respective open-label extension studies (CL0600-005 and STEPS-2/STEPS-3, completed in 2010 and 2013) [18–20]. Among patients who continued treatment in the open-label extension studies, a greater proportion (8–17%) achieved enteral autonomy with long-term treatment than with shorter term treatment [16–20]. A similar efficacy was observed in a 12-week pediatric study (TED-C13-003), a 24-week pediatric study (TED-C14-006), and their respective open-label extension studies (SHP633-303 and SHP633-304).

The benefits and risks associated with teduglutide in Japanese patients with SBS-IF have not been previously reported. We therefore report herein the efficacy, safety, and pharmacokinetic (PK) findings for teduglutide from two multicenter phase III clinical trials and their extension study in adult Japanese patients with SBS-IF: the up to 30-month TED-C14-004 study; the 24-week SHP633-306 pivotal study; and the SHP633-307 long-term extension study (involving ≥ 12 months’ and 24 weeks’ participation in studies TED-C14-004 and SHP633-306, respectively). These three studies were conducted with the goal of achieving marketing approval for teduglutide in Japan.

## Methods

### Study design

Two open-label, multicenter phase III clinical trials of teduglutide in adult patients with SBS-IF and their extension study were conducted in Japan: TED-C14-004 (ClinicalTrials.gov identifier NCT02340819) involved short-term (24 weeks) and long-term (up to 30 months) treatment with teduglutide; SHP633-306 (ClinicalTrials.gov identifier NCT03663582) involved only short-term (24 weeks) treatment with teduglutide; and SHP633-307 (ClinicalTrials.gov identifier NCT03596164) was a long-term extension study that enrolled patients who completed TED-C14-004 or SHP633-306.

TED-C14-004 was conducted between December 2014 and November 2018, and SHP633-306 was conducted between July 2018 and August 2019. SHP633-307 is ongoing at the time of this analysis, so February 2020 was used as the interim data cut-off point. The studies were conducted in accordance with the principles of the World Medical Association Declaration of Helsinki and applicable laws and standards of good clinical practice in Japan. Upon approval of the study protocol from institutional review boards or ethics committees, written informed consent from patients was obtained prior to their involvement in any study activities. The inclusion and exclusion criteria, dose of teduglutide, and treatment duration used for studies TED-C14-004 and SHP633-306 were based on those used in the pivotal multinational STEPS-1 study [17].

Patients who entered TED-C14-004 or SHP633-306 had to be ≥ 16 years old, have SBS as a result of major intestinal resection, and have been dependent on PS ≥ 3 times per week for ≥ 12 months before enrollment, with a < 10% change in weekly PS volume for ≥ 4 consecutive weeks before the start of treatment with teduglutide. Patients with a history of Crohn’s disease could participate if they had been in clinical remission (inactive Crohn’s disease demonstrated by clinical assessment) for ≥ 12 weeks before teduglutide dosing. Key exclusion criteria included use of native GLP-2 or human growth hormone in the 6 months before study start or any prior use of teduglutide. To be eligible for SHP633-307, patients had to have completed SHP633-306 or had ongoing participation in TED-C14-004 at the time of study closure.

**Fig. 1:**
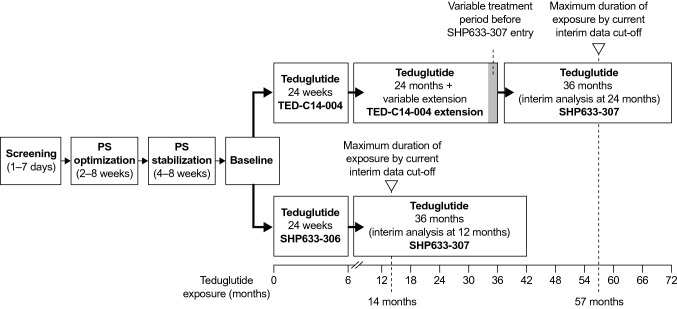
Study design of TED-C14-004, SHP633-306, and SHP633-307. After completing the period of PS optimization and stabilization, patients received teduglutide 0.05 mg/kg subcutaneously once daily for up to 24 weeks (SHP633-306), 30 months (TED-C14-004), or 36 months (SHP633-307). PS volume adjustments during the teduglutide dosing period were based on changes from the baseline urine output and other assessments of fluid balance and nutrition status. *PS*, parenteral support; *SE*, standard error

An overview of the study designs for TED-C14-004, SHP633-306, and SHP633-307 is given in Fig. 1. As in the previous STEPS-1 study, patients participating in the core studies (TED-C14-004 and SHP633-306) underwent an optimization (2–8 weeks) and stabilization (4–8 weeks) period before initiation of treatment with teduglutide, during which PS was adjusted with the goal of achieving a stable target urine output of 1.0–2.0 L/day [17]. All participating patients received teduglutide 0.05 mg/kg SC once daily throughout these three studies. PS was adjusted according to an algorithm based on the change from baseline in urine output and other assessments of fluid balance and nutrition status; the PS adjustment algorithm required reductions in PS if the urine output rose more than 10% above baseline. Patients had study visits at weeks 2 and 4, after which visits were held monthly during TED-C14-004 and SHP633-306. During SHP633-307, patients from SHP633-306 had monthly study visits throughout the 24 total months of treatment and then at 3-month intervals thereafter, and patients from TED-C14-004 had visits at 3-month intervals. The end-of-study assessments for SHP633-306 and TED-C14-004 were combined with the first visit of SHP633-307. SHP633-307 is ending soon with the approval of teduglutide in Japan [15].

In TED-C14-004, there was no training for patients or caregivers on how to administer teduglutide at home. However, for SHP633-306 and SHP633-307, patients or caregivers were provided a detailed training manual and a quick reference guide. The first dose was administered by a study physician, who then observed the patient or caregiver administering teduglutide at least twice in compliance with an administration checklist prior to patients or caregivers administering the drug unsupervised. To ensure teduglutide continued to be administered correctly and safely at home, the study physician supervised administration at select study visits during the treatment period. To facilitate the preparation and administration of teduglutide, a vial adaptor was also implemented in SHP633-306 and SHP633-307 for reconstitution of lyophilized teduglutide with sterile water for injection and drawing of the reconstituted drug into a dosing syringe with a safety lock.

### Data collection

For TED-C14-004 and SHP633-306, patient demographic and clinical characteristics were captured at baseline, defined as the last visit before initiation of treatment with teduglutide. The baseline for the analysis of the efficacy and PK assessments for each patient was the baseline from core studies TED-C14-004 or SHP633-306. Data collected at each clinic visit included the daily PS volume, safety parameters, clinical laboratory test results, vital signs, body weight, and body mass index (BMI).

Concentrations of plasma citrulline, a biomarker of intestinal epithelial mass, were measured at baseline; weeks 4, 8, 16, and 24; and then every 2 months thereafter up to 30 months of treatment. Patients who continued into SHP633-307 from SHP633-306 had measurements taken at month 12, while those continuing from TED-C14-004 had measurements taken every 3 months.

In TED-C14-004 and SHP633-306, blood samples for PK evaluations were taken pre-dose and at 0.25, 0.5, 1, 2, 3, 4, 6, 8, 10, and 12 h post-dose during the first 12 weeks of treatment with teduglutide. In SHP633-307, patients continuing from study TED-C14-004 had blood samples for PK evaluations taken pre-dose and at 0.25 and 0.5 min, and 1, 2, 3, 4, 6, 8, 10, and 12 h post-dose at the first extension study visit. Patients continuing from SHP633-306 had blood samples taken pre-dose and 1 and 2 h post-dose at the first extension study visit. Blood samples to test for the presence of antibodies to teduglutide were taken at baseline and weeks 12 and 24 in SHP633-306; ≥ 14 h after dosing at baseline and weeks 12 and 24, and then every 6 months thereafter in TED-C14-004; and every 3 months in SHP633-307.

### Data analyses

The intent-to-treat (ITT) population consisted of patients who enrolled and were eligible at baseline (i.e., after PS optimization and stabilization) to receive teduglutide. Efficacy analyses were based on the ITT population, all of whom received teduglutide. The safety population comprised all patients in the ITT population who received ≥ 1 dose of teduglutide and the PK population included all patients in the safety analysis population for whom PK data were sufficient and interpretable. In these studies, the ITT population was the same as the safety population. Treatment compliance was defined as patients receiving ≥ 80% of the planned study drug doses, assessed by site investigators counting used/unused vials at every clinic visit.

Efficacy endpoints were defined in an analogous manner to previous pivotal studies [17, 19, 20]. These included a clinically meaningful reduction in weekly PS volume of ≥ 20% [17] measured between baseline and both 20 and 24 weeks for TED-C14-004 and SHP633-306, and a continued reduction in weekly PS volume of ≥ 20% from the baseline in core studies at each scheduled visit until the last visit before the interim data cut-off of SHP633-307. Other efficacy endpoints included changes in absolute and percentage weekly PS volumes from the baseline in the core studies at each study visit, the number of days per week of PS, the number of patients who achieved enteral autonomy at the end of treatment or the data cut-off, and the changes in plasma citrulline levels from baseline in the core studies. The primary efficacy data were derived from patient diaries and compared against investigator-prescribed data captured in electronic case report forms.

Efficacy was evaluated between baseline and 24 weeks in TED-C14-004 and SHP633-306 and between baseline and 30 months in TED-C14-004. Among patients who entered SHP633-307, the total follow-up periods, including the prior studies, were up to 14–57 months of total teduglutide exposure for patients from SHP633-306 and TED-C14-004.

Post hoc analyses of PS volume reductions in patients with Crohn’s disease, with and without a stoma, were performed for TED-C14-004.

Single-dose PK parameters were estimated using a non-compartmental analysis and included the maximum plasma concentration of teduglutide (C_max_), time to C_max_ (t_max_), terminal half-life elimination (t_½_), area under the plasma concentration–time curve (AUC) from time 0 to infinity (AUC_0-inf_), and AUC from time 0 to the last measurable concentration (AUC_0-t_).

Safety analyses included the monitoring of adverse events (AEs), body weight, BMI, vital signs, 12-lead electrocardiogram (ECG), serum biochemistry, hematology, and presence of antibodies to teduglutide. AEs were coded using the Medical Dictionary for Regulatory Activities versions 17.1 (TED-C14-004) and 21.0 (SHP633-306 and SHP633-307) [21]. Specifically, AEs were treatment-emergent AEs (TEAEs), defined as any unfavorable and unintended sign (including any abnormal laboratory finding), symptom, or disease that occurred during treatment with teduglutide, whether or not the event was considered to be related to the study drug by the clinical study investigator. AEs were also categorized as mild, moderate, or severe. Treatment-emergent serious AEs (TESAEs) were defined as any medical occurrence that required hospitalization, resulted in persistent or significant disability, resulted in a congenital abnormality or birth defect, was life-threatening, resulted in death, or was an important medical event in the opinion of the investigator. AEs were considered related to treatment with teduglutide, or its administration, based on the investigator's medical judgment.

### Statistical analyses

The small size of the study populations precluded any formal statistical testing; data were summarized using descriptive statistics. All data values are reported as the mean ± standard deviation (SD) unless otherwise stated.

## Results

### Patients

Patient demographics and baseline clinical characteristics are summarized in Table 1. Crohn’s disease was the most common primary reason for intestinal resection. Prior to starting teduglutide treatment, patients in TED-C14-004 had a mean duration of SBS roughly a third of the length of time of those in SHP633-306 (6.6 vs 19.3 years) and had been dependent on PS for almost half as long (5.2 vs 11.1 years). Patients in TED-C14-004 had a greater proportion of colon remaining (61.7 ± 31.3%) than patients in SHP633-306 (31.3 ± 14.4%) but conversely had a shorter small intestine (102.7 ± 80.4 cm) than patients in SHP633-306 (233.4 ± 133.5 cm). One patient (9.1%) in TED-C14-004 had a terminal ileum; no patients in SHP633-306 had a terminal ileum. The mean PS volume at baseline was 17.3 ± 8.8 L/week in TED-C14-004 and 14.9 ± 6.8 L/week in SHP633-306. For patients who continued into SHP633-307, the PS volume at baseline in the core studies was 15.7 ± 8.7 L/week from TED-C14-004 and 13.0 ± 5.5 L/week for patients from SHP633-306.

**Table 1:** Patient demographics and baseline clinical characteristics

Parameter	TED-C14-004 (*N* = 11)	SHP633-306 (*N* = 7)	SHP633-307^a^		
			TED-C14-004 (*N* = 7)	SHP633-306 (*N* = 4)	Total (*N* = 11)
Age, years, mean (SD)	40.9 (12.4)	40.4 (8.9)	42.4 (7.2)	42.4 (7.2)	41.8 (8.8)
Age group, years, *n* (%)					
16 to < 45	7 (63.6)	5 (71.4)	4 (57.1)	2 (50.0)	6 (54.5)
45 to < 65	4 (36.4)	2 (28.6)	3 (42.9)	2 (50.0)	5 (45.5)
Sex, *n* (%)					
Male	8 (72.7)	3 (42.9)	5 (71.4)	2 (50.0)	7 (63.6)
Ethnicity, Japanese, n (%)	11 (100.0)	7 (100.0)	7 (100.0)	4 (100.0)	11 (100.0)
Height, cm, mean (SD)	167.1 (7.6)	160.5 (5.8)	167.7 (6.7)	161.1 (6.1)	165.4 (7.1)
Weight, kg, mean (SD)	55.9 (8.7)	48.9 (7.1)	56.0 (11.0)	49.3 (10.0)	53.6 (10.7)
BMI, kg/m^2^, mean (SD)	20.0 (2.7)	19.0 (2.6)	19.8 (3.2)	18.9 (3.4)	19.5 (3.1)
Duration of SBS, years, mean (SD)	6.6 (3. 6)	19.3 (6.8)	7.07 (3.0)	22.41 (4.5)	12.65 (8.5)
Primary reason for intestinal resection, *n* (%)					
Crohn’s disease^b^	8 (72.7)	6 (85.7)	6 (85.7)	3 (75.0)	9 (81.8)
Volvulus	2 (18.2)	1 (14.3)	1 (14.3)	1 (25.0)	2 (18.2)
Graft rejection after small bowel transplantation	1 (9.1)	0	0	0	0
Patients with stoma, *n* (%)	6 (54.5)	5 (71.4)	5 (71.4)	2 (50.0)	7 (63.6)
Type of stoma, *n* (%)					
Ileostomy	5 (83.3)	5 (100.0)	4 (80.0)	2 (100.0)	6 (85.7)
Jejunostomy	1 (16.7)	0	1 (20.0)	0	1 (14.3)
Patients with remaining colon, *n* (%)	6 (54.5)	4 (57.1)	3 (42.9)	3 (75.0)	6 (54.5)
Estimated percentage of colon remaining, mean (SD)	61.7 (31.3)	31.3 (14.4)	60.7 (36.9)	36.7 (11.6)	48.7 (27.8)
Estimated length of small intestine, cm, mean (SD)	102.7 (80.4)	233.4 (133.5)	112.1 (83.7)	320 (81.9)	174.5 (127.42)
Patients with distal/terminal ileum, *n* (%)	1 (9.1)	0	0	0	0
Duration of PS dependency, years, mean (SD)	5.2 (3.6)	11.1 (3.9)	7.07 (3.0)	22.41 (4.5)	12.65 (8.5)
PS volume, L/week, mean (SD)	17.3 (8.8)	14.9 (6.8)	15.7 (8.7)	13.0 (5.5)	14.7 (7.5)
Number of days/week of PS, mean (SD)	7.0 (0)	7.0 (0.0)	7.0 (0.0)	7.0 (0.0)	7.0 (0.0)

*BMI* body mass index, *PS* parenteral support, *SBS* short bowel syndrome, *SD* standard deviation

^a^Patients in SHP633-307 had continued from either TED-C14-004 or SHP633-306.

^b^All patients with Crohn’s disease were in clinical remission

In TED-C14-004, 2 patients discontinued during 24 weeks of treatment: 1 due to a TEAE of acute liver failure and 1 after withdrawing their consent. Another patient withdrew consent after completing 24 weeks of treatment because of diarrhea; this patient did not respond to teduglutide. One patient discontinued due to withdrawn consent during 24 months of treatment. In SHP633-306, 6 patients completed 24 weeks of treatment, and 1 discontinued treatment due to their physician’s decision. Eleven patients enrolled in SHP633-307, including 7 from TED-C14-004 and 4 from SHP633-306. There was no interruption in treatment when patients transitioned to SHP633-307. At the interim data cut-off of SHP633-307, all 11 patients continued receiving teduglutide.

### Mean exposure to teduglutide

Patients who completed the extension study until the interim data cut-off point received teduglutide up to a maximum of 14 months (SHP633-306) or 57 months (TED-C14-004). The mean extents of teduglutide exposure only in TED-C14-004 and SHP633-306 were 26.8 ± 17.0 months and 4.8 ± 1.6 months, respectively. The mean cumulative extents of exposure to teduglutide for patients who completed both the core and the extension study treatments were 54.3 ± 1.7 and 12.7 ± 1.0 months for patients from TED-C14-004 and SHP633-306, respectively.

### Efficacy

**Fig. 2:**
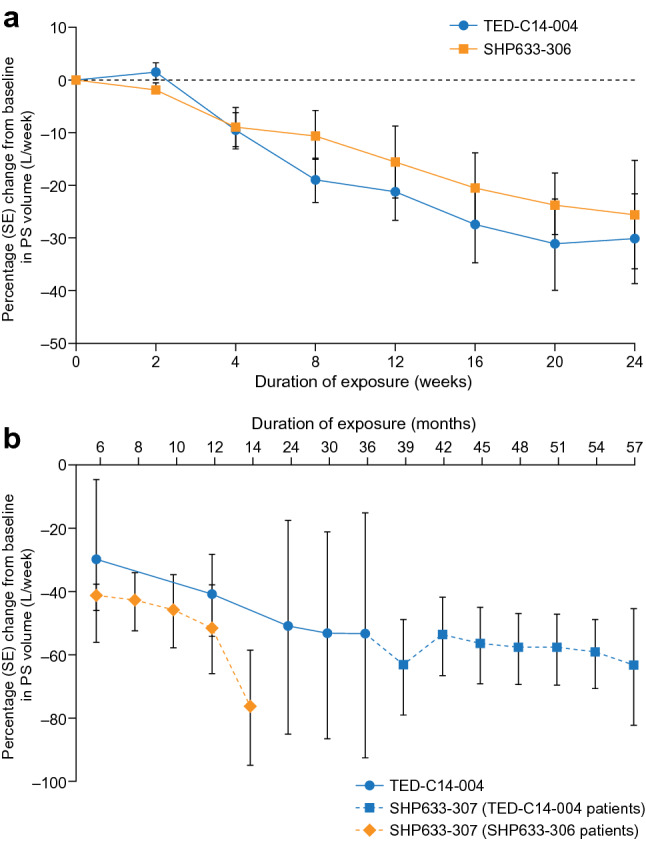
Mean percent change in the PS volume from baseline up to 24 weeks (**a**) and beyond 24 weeks (**b**) of treatment with teduglutide. The week 24 time point for SHP633-306 (*n* = 6) differs from the month 6 time point for SHP633-307 (SHP633-306 patients, *n* = 4) due to differences in the number of patients participating in each study. *PS*, parenteral support; *SE*, standard error

- Short-term treatment with teduglutide
  The first 24 weeks of treatment with teduglutide were considered short-term use.
- Change in PS requirements
  A reduction in weekly PS volume of ≥ 20% from baseline at both weeks 20 and 24 was achieved by 9 of 18 patients (50%) (5 of 11 patients [45.5%] in TED-C14-004 and 4 of 7 patients [57.1%] in SHP633-306). At 24 weeks, the PS volume decreased from baseline by 4.9 ± 4.8 L/week (−30.1% ± 25.9%; *n* = 9) and 3.3 ± 3.6 L/week (−25.6% ± 25.5%; *n* = 6) for patients from studies TED-C14-004 and SHP633-306, respectively (Fig. 2a, Online Resource 1a). Patients’ mean reduction in PS infusion frequency also decreased by 1.0 ± 1.8 days/week (*n* = 9; TED-C14-004) and 0.5 ± 1.23 days/week (*n* = 6; SHP633-306) from baseline. No patients achieved enteral autonomy during the first 24 weeks of receiving teduglutide (SHP633-306 and TED-C14-004).
- Change in plasma citrulline level
  At 24 weeks, the level of plasma citrulline increased from baseline by 15.0 ± 17.4 µM (86.9% ± 112.3%, *n* = 9) and 14.3 ± 10.9 µM (106.3% ± 114.9%, *n* = 6) for patients in TED-C14-004 and SHP633-306, respectively (Online Resource 2).
- Urine output
  At 24 weeks, the 48 h urine output increased from a baseline of 2933.0 ± 563.0 mL/day (*N* = 11) by 226.7 ± 555.1 mL/day (9.3% ± 22.4%; *n* = 9) for patients from TED-C14-004. In SHP633-306, at 24 weeks, the 48 h urine output increased from a baseline of 1255.0 ± 268.1 mL/day (*N* = 7) by 212.5 ± 265.9 mL/day (18.0% ± 23.9%; *n* = 6).
- Long-term treatment with teduglutide
  Patients who participated in TED-C14-004 received up to 30 months of treatment. By the interim cut-off point of the extension study (SHP633-307), patients from SHP633-306 and TED-C14-004 received a maximum of 14 and 57 months of treatment, respectively.
- Change in PS requirements
  All patients who participated in the extension study following SHP633-306 maintained  ≥ 20% reduction from baseline at each scheduled visit to the last visit before the data cut-off. After 30 months of treatment, 6 of 7 patients (85.7%) in TED-C14-004 achieved  ≥ 20% reduction in weekly PS volume from baseline. By the end of TED-C14-004, all patients had achieved  ≥ 20% reduction in weekly PS volume, which was maintained to the last visit before the data cut-off in the extension study.
  Reductions in PS volume continued with long-term treatment with teduglutide. Patients from SHP633-306 had notable and progressive reductions in PS from baseline; at 12 months, the PS volume had decreased by 7.3 ± 6.5 L/week (−51.6% ± 28.5%; *n* = 4). By the last visit before the interim data cut-off of SHP633-307, PS for patients from SHP633-306 had reduced by 11.7 ± 8.6 L/week (−76.4% ± 25.9%; *n* = 2) from baseline. At 30 months (TED-C14-004), the PS volume had decreased by 8.3 ± 5.9 L/week (−53.5% ± 33.9%; *n* = 7) from baseline (Fig. 2b, Online Resource 1b). For patients from TED-C14-004 who advanced to the extension study, at 39 months, the PS volume had decreased by 7.9 ± 4.5 L/week (−63.6% ± 33.9%; *n* = 5), and at the interim data cut-off, the PS volume had decreased by 11.5 ± 3.7 L/week (−63.57% ± 32.0%; *n* = 3). No change in efficacy was observed when switching to the vial adaptor for teduglutide administration for patients in TED-C14-004. With long-term treatment, patients also experienced overall reductions in the number of days per week that they required PS. By the interim data cut-off of the extension study, the PS infusion frequency had decreased from baseline in the core study by 1.9 ± 2.3 days/week for patients from SHP633-306. After 30 months of receiving teduglutide, the mean PS infusion frequency had decreased by 2.1 ± 3.3 days/week (*n* = 7; TED-C14-004), remaining relatively stable up to the end of the extension study, with a reduction of 2.2 ± 3.3 days/week for patients from baseline of TED-C14-004.
  In TED-C14-004, 2 of 8 patients (25%) achieved enteral autonomy by 30 months of treatment. Both had Crohn’s disease recorded as the reason for small bowel resection, an ileostomy, no remaining colon, and a remaining small intestinal length of 100 cm in one and 120 cm in the other. Furthermore, both remained off PS throughout SHP633-307 until its interim data cut-off. During SHP633-307, 1 patient from SHP633-306 almost achieved enteral autonomy at the interim data cut-off point, which occurred 14 months after this patient had begun treatment. Based on diary data, this individual received 0.5 L/day of PS for 2 days/week during the 2 weeks prior to their visit at month 14, but did not receive PS during the 7 days following this visit.
- Change in plasma citrulline level
  The levels of plasma citrulline continued to increase with long-term treatment, showing an increase of 13.0 ± 5.6 µM (79.1% ± 33.5%) from baseline for patients from SHP633-306 at the interim cut-off of the extension study (Online Resource 2). For patients in TED-C14-004, the plasma citrulline level continued to increase through 24 months of treatment and plateaued thereafter. After 30 months of treatment, the increase from baseline was 28.3 ± 24.1 µM (135.0% ± 84.2%; *n* = 7). Only slight additional changes were observed by the interim data cut-off of SHP633-307; in particular, the plasma citrulline level had increased by 19.2 ± 18.9 µM (103.6% ± 88.7%) compared with baseline for patients from TED-C14-004 (up to 57 months; *n* = 7).
- Urine output

For patients who advanced to SHP633-307 from SHP633-306, further increases from baseline (1242.5 ± 374.1 mL/day; *N* = 4) were seen after 6 months (345.0 ± 217.8 mL/day; 29.0 ± 21.6%; *n* = 4) and after 12 months (1592.5 ± 806.0 mL/day; *n* = 4). At 30 months (TED-C14-004), the urine output had increased by 306.0 ± 735.0 mL/day (11.2% ± 24.6%; *n* = 7) from the baseline value of 2933.0 ± 563.0 mL/day (*N* = 11). In patients who advanced from TED-C14-004 to the extension study, the mean urine output from baseline (1430.0 ± 285.4 mL/day; *N* = 7) remained relatively stable, reaching an output of 1388.2 ± 507.8 mL/day (−2.0% ± 35.3%; *n* = 7) at the last visit before the interim data cut-off of SHP633-307.

### Post hoc analyses of TED-C14-004

Post hoc analyses of TED-C14-004 were performed for both short- and long-term treatment of patients in this study. In the subgroup of patients with Crohn’s disease, the mean baseline PS volume requirement was 16.3 ± 8.4 L/week (*n* = 8). After 24 weeks and 30 months of treatment with teduglutide, the PS volume decreased by 5.3 ± 5.4 L/week (−31.9% ± 28.9%; *n* = 7) and 9.3 ± 5.8 L/week (−59.4% ± 32.9%; *n* = 6), respectively. In the subgroup of patients with a stoma, the baseline PS volume was 18.3 ± 8.5 L/week (*n* = 6). After 24 weeks and 30 months of treatment, the PS volume decreased by 6.1 ± 5.5 L/week (−35.3% ± 30.1%; *n* = 6) and 10.7 ± 5.4 L/week (−65.2% ± 33.2%; *n* = 5), respectively. In patients without a stoma, the baseline PS volume was 15.9 ± 7.1 L/week (*n* = 5). After 24 weeks and 30 months of treatment, the PS volume decreased by 2.7 ± 1.8 L/week (−19.6% ± 13.1%; *n* = 3) and 2.5 ± 0.2 L/week (−24.3% ± 8.7%; *n* = 2), respectively.

### PK analyses

In TED-C14-004, data for PK analyses were available for 8 patients (72.7%); teduglutide was undetectable in the plasma of the other 3 patients at all time points, most likely because of dosing errors (improved training of patients or caregivers to administer teduglutide was then implemented in SHP633-306 and SHP633-307). PK analyses of teduglutide showed that a C_max_ ± SD of 49.7 ± 19.7 ng/mL was achieved at t_max_ ± SD 2.6 ± 1.0 h after daily dosing, and teduglutide was eliminated from plasma with a mean t_½_ ± SD of 1.2 ± 0.7 h (*n* = 8). In addition, the mean ± SD AUC_0-t_ and AUC_0-inf_ values were 201 ± 51.6 and 204 ± 51.1 ng⋅h/mL, respectively. Similarly, in SHP633-306, a C_max_ ± SD of 49.5 ± 16.4 ng/mL was achieved at a t_max_ ± SD of 3.6 ± 1.6 h post-dose (*n* = 7), and teduglutide was eliminated from plasma with a mean t_½_ ± SD of 1.1 ± 0.2 h (*n* = 6). The mean ± SD AUC_0–t_ and AUC_0–inf_ values for plasma teduglutide were 240 ± 80.7 and 252 ± 85.4 ng⋅h/mL, respectively (*n* = 7).

Patients in SHP633-307 from TED-C14-004 achieved a C_max_ ± SD of 54.1 ± 18.7 ng/mL at a t_max_ ± SD of 2.1 ± 0.4 h post-dose. Teduglutide was eliminated from plasma with a mean t_½_ of 1.3 ± 0.6 h. The mean ± SD AUC_0–t_ and AUC_0–inf_ values for plasma teduglutide were 200.4 ± 71.6 ng⋅h/mL and 208.7 ± 72.7 ng⋅h/mL, respectively. For patients in SHP633-307 from SHP633-306, sparse PK sample collection and population PK modeling were used, and all pre-dose concentrations after multiple dosing were below the lower limit quantification, suggesting no accumulation of teduglutide.

### Safety

There were no clinically meaningful changes during any of the studies concerning the clinical laboratory measures, vital signs, body weight, BMI, or ECG findings. There were also no AEs related to growth of pre-existing polyps of the colon or gastrointestinal cancers observed during the studies.

### TEAEs

The majority of all TEAEs reported were mild or moderate in severity. During the first 24 weeks of treatment (TED-C14-004 and SHP633-306), TEAEs reported in ≥ 2 patients were recorded for 17 (94.4%) patients with a total of 121 events (Table 2). The most frequently reported TEAEs were gastrointestinal disorders, including abdominal pain and abdominal distension. Infections and infestations were also regularly reported, of which device-related infection (e.g., central line infection) was the most frequently reported. Beyond the first 24 weeks of treatment (TED-C14-004 and SHP633-307), 199 TEAEs were reported by 11 (73.3%) patients. The most frequently reported issues were infections and infestations, including device-related infection. Gastrointestinal disorders, including diarrhea and nausea, were still among the most frequently reported TEAEs. TEAEs considered to be related to teduglutide treatment and reported in ≥ 2 patients were abdominal distension, abdominal pain, injection-site reaction, pyrexia, and vomiting.

**Table 2:** Summary of TEAEs reported in at least two teduglutide-treated patients

System organ class and preferred term	≤ 24 weeks (SHP633-306 and TED-C14-004)		> 24 weeks (SHP633-307 and TED-C14-004)		Total	
	Patients *n* (%)	Events *n*	Patients *n* (%)	Events *n*	Patients *n* (%)	Events *n*
Any TEAE	17 (94.4)	121	11 (73.3)	199	18 (100.0)	320
Blood and lymphatic system disorders	3 (16.7)	4	0	0	3 (16.7)	4
Anemia	2 (11.1)	2	0	0	2 (11.1)	2
Eye disorder	2 (11.1)	2	1 (6.7)	5	3 (16.7)	7
Gastrointestinal disorders	12 (66.7)	27	8 (53.3)	33	15 (83.3)	60
Abdominal distension	4 (22.2)	4	2 (13.3)	2	6 (33.3)	6
Abdominal pain	5 (27.8)	5	1 (6.7)	1	5 (27.8)	6
Diarrhea	1 (5.6)	1	3 (20.0)	4	4 (22.2)	5
Enteritis	1 (5.6)	1	1 (6.7)	1	2 (11.1)	2
Enterocolitis	1 (5.6)	2	2 (13.3)	2	3 (16.7)	4
Nausea	1 (5.6)	1	3 (20.0)	7	4 (22.2)	8
Stomatitis	1 (5.6)	1	3 (20.0)	3	4 (22.2)	4
Vomiting	2 (11.1)	2	1 (6.7)	1	3 (16.7)	3
General disorders and administration site conditions	9 (50.0)	21	5 (33.3)	16	10 (55.6)	37
Catheter site pain	1 (5.6)	1	2 (13.3)	4	2 (11.1)	5
Device occlusion^a^	2 (11.1)	3	2 (13.3)	3	3 (16.7)	6
Injection site reaction	4 (22.2)	4	0	0	4 (22.2)	4
Pyrexia	6 (33.3)	6	4 (26.7)	5	8 (44.4)	11
Hepatobiliary disorders	2 (11.1)	3	2 (13.3)	2	4 (22.2)	5
Immune system disorders	0	0	2 (13.3)	2	2 (11.1)	2
Hypersensitivity	0	0	2 (13.3)	2	2 (11.1)	2
Infections and infestations	8 (44.4)	19	10 (66.7)	62	12 (66.7)	81
Anal abscess	0	0	3 (20.0)	3	3 (16.7)	3
Catheter site infection	1 (5.6)	1	1 (6.7)	1	2 (11.1)	2
Device-related infection^a^	6 (33.3)	9	7 (46.7)	24	10 (55.6)	33
Influenza	0	0	5 (33.3)	18	7 (38.9)	21
Nasopharyngitis	3 (16.7)	3	5 (33.3)	18	7 (38.9)	21
Oral herpes	1 (5.6)	1	1 (6.7)	1	2 (11.1)	2
Injury, poisoning, and procedural complications	4 (22.2)	5	1 (6.7)	1	5 (27.8)	6
Investigations	6 (33.3)	9	2 (13.3)	5	7 (38.9)	14
Metabolism and nutrition disorders	4 (22.2)	6	5 (33.3)	7	7 (38.9)	13
Dehydration	2 (11.1)	2	2 (13.3)	2	2 (11.1)	4
Hypozincemia	1 (5.6)	1	1 (6.7)	1	2 (11.1)	2
Musculoskeletal and connective tissue disorders	2 (11.1)	2	6 (40.0)	10	8 (44.4)	12
Arthralgia	0	0	5 (33.3)	5	5 (27.8)	5
Back pain	1 (5.6)	1	1 (6.7)	1	2 (11.1)	2
Nervous system disorders	4 (22.2)	7	7 (46.7)	19	8 (44.4)	26
Headache	3 (16.7)	3	5 (33.3)	11	7 (38.9)	14
Somnolence	0	0	3 (20.0)	3	3 (16.7)	3
Psychiatric disorders	0	0	3 (20.0)	4	3 (16.7)	4
Insomnia	0	0	2 (13.3)	2	2 (11.1)	2
Renal and urinary disorders	2 (11.1)	2	3 (20.0)	4	5 (27.8)	6
Hematuria	1 (5.6)	1	2 (13.3)	2	3 (16.7)	3
Respiratory, thoracic, and mediastinal disorders	5 (27.8)	5	3 (20.0)	8	7 (38.9)	13
Oropharyngeal pain	1 (5.6)	1	3 (20.0)	3	4 (22.2)	4
Upper respiratory tract inflammation	2 (11.1)	2	1 (6.7)	1	2 (11.1)	3
Skin and subcutaneous tissue disorders	4 (22.2)	5	7 (46.7)	13	9 (50.0)	18
Dermatitis contact	0	0	4 (26.7)	4	4 (22.2)	4
Dry skin	1 (5.6)	1	1 (6.7)	1	2 (11.1)	2
Hemorrhage subcutaneous	1 (5.6)	1	2 (13.3)	2	2 (11.1)	3
Pruritus	1 (5.6)	1	1 (6.7)	1	2 (11.1)	2
Vascular disorders	2 (11.1)	2	3 (20.0)	4	4 (22.2)	6
Vascular pain	0	0	2 (13.3)	3	2 (11.1)	3

The majority of TEAEs were single events in individual patients

*PS*, parenteral support, *TEAE*, treatment-emergent adverse event

^a^All device-related infection and device occlusion events were related to the central venous catheter for PS administration and not to the teduglutide injection device

Both during and after the first 24 weeks of treatment, the majority of TESAEs were single events, and the most frequently reported were device-related infection. All device-related infection events were related to central venous catheters used to administer PS and not to the teduglutide injection device. No TESAEs reported in SHP633-306 or SHP633-307 were considered to be related to teduglutide. Four TESAEs reported in 2 patients (18.2%) in TED-C14-004 were considered by the investigator to be related to treatment with teduglutide. Severe pyrexia was reported in 1 patient, and acute hepatic failure, enterocolitis, and intestinal obstruction were reported in a different patient; the latter patient had a history of congenital microvillus inclusion disease, SBS-IF, and chronic liver disease at study baseline. That patient developed the aforementioned TESAEs within 14 days of the first dose of teduglutide. Treatment with teduglutide was discontinued, and this individual died of acute hepatic and renal failure 52 days after teduglutide discontinuation. This was the only death that occurred during the study. The investigator reported only the event of acute hepatic failure as related to the administration of teduglutide.

#### Anti-teduglutide antibodies

**Fig. 3:**
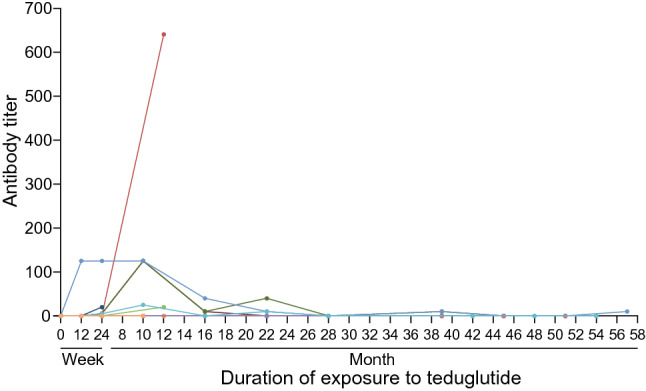
Detection of anti-teduglutide antibodies from baseline to the interim data cut-off point

At 24 weeks, 5 out of 14 patients (35.7%; *n* = 4, TED-C14-004; *n* = 1, SHP633-306) tested positive for anti-teduglutide antibodies (Fig. 3); of those, 1 patient also tested positive for neutralizing antibodies. Three of the 4 patients in TED-C14-004 who tested positive for anti-teduglutide antibodies at 24 weeks also tested positive up to 22 months. Of the patients who continued into the extension study (SHP633-307) from SHP633-306, 2 out of 4 (50.0%) tested positive for antibodies to teduglutide at month 12, the final antibody assessment before the interim data cut-off; 1 of those patients also tested positive for neutralizing antibodies. Two patients (40.0%) who continued from TED-C14-004 into SHP633-307 had low titers of anti-teduglutide antibodies as well as neutralizing antibodies detected at 39 months. No anti-teduglutide antibodies were detected at 51 or 54 months in any patients (*n* = 7). A low titer of anti-teduglutide antibodies was detectable in 1 patient at 57 months.

The patient with the highest titer of anti-teduglutide antibodies (1:650 at month 12) had sustained reductions in PS volume at the time of the interim data cut-off (28.6% reduction in PS volume), and there were no signs of hypersensitivity. Among the 7 patients who tested positive for anti-teduglutide antibodies, 1 (14.3%) did not achieve a reduction in PS volume greater than 20% and discontinued treatment after week 24; this patient had a low titer of anti-teduglutide antibodies (1:20) at week 24. Overall, no association with AEs of hypersensitivity or teduglutide-related hypersensitivity reaction was observed among the patients in whom anti-teduglutide antibodies were detected. Furthermore, no association between anti-teduglutide antibodies and a lack of efficacy was observed.

## Discussion

Teduglutide is the first and only GLP-2 analogue currently available worldwide for treatment of SBS-IF [22]. The two phase III and extension studies reported here evaluated the efficacy, safety, and PK profile of teduglutide 0.05 mg/kg SC once daily in the short- and long-term treatment of adult Japanese patients with SBS-IF. Our observations include the longest duration of treatment with teduglutide reported for clinical trials in the literature to date and the first set of teduglutide studies focused on Japanese patients with SBS-IF.

The efficacy and safety profile of teduglutide observed in Japanese patients was generally consistent across these studies as well as with the previously reported values in pivotal multinational STEPS studies [17, 19, 20], despite some differences in baseline demographics and characteristics. Notable differences in baseline parameters among TED-C14-004, SHP633-306, and STEPS-1 were in the mean residual length of small intestine, proportion of patients with Crohn’s disease, and proportion of patients with an ileostomy who had a stoma [17]. In addition, compared with STEPS-1, patients in TED-C14-004 and SHP633-306 had a lower mean weight and BMI and higher mean PS volumes and days per week of PS at baseline. These differences carried through into the extension studies. The unusually long residual small bowel length and long duration of IF among patients suggest that these patients had an impaired function of the residual small bowel due to ongoing inflammation or irreversible sequelae of chronic damage [19, 20]. Furthermore, the differences observed may have been related to hypofunction of the residual intestine in Crohn’s disease or the high volume of intestinal fluid drainage associated with stoma construction. Although mesenteric ischemia is a frequent cause of SBS [23–25], in the current studies in Japanese patients, Crohn’s disease was the most common cause of SBS. In Crohn’s disease, the small bowel function can be impaired even when the length is not affected, due to reasons such as inflammation of stricture in the intestines [26, 27]. There are currently limited data on the efficacy of teduglutide in patients with both SBS-IF and Crohn’s disease; however, teduglutide has previously been found to be safe and effective at reducing PS requirements in patients with these combined conditions [28]. This finding is supported by the identification of a similar percentage change in PS volume at 24 weeks and a slightly greater reduction in PS volume at 30 months for patients with Crohn’s disease compared with the overall patient response in TED-C14-004.

As well as differing in disease characteristics, patients in these studies were all Japanese, whereas those who participated in the STEPS international phase III studies conducted in Europe and North America were mostly Caucasian. Differences in race or ethnicities can result in variabilities in the efficacy, PK, and/or safety of some therapies; these variations can be attributed to differences in certain aspects, such as physiological and socio-cultural factors [29, 30]. Reductions in the PS volume after 24 weeks of treatment in this study were similar to those observed in STEPS-1 [17, 31] and were also sustained during longer term treatment, which is consistent with STEPS-2 and STEP-3 [19, 20]. The plateau in PS reductions after 30 months of treatment is also consistent with STEPS-3 [20] and suggests that it takes an average of over 2 years for the physiologic effects of teduglutide to peak, which supports its long-term use to treat patients with SBS-IF. No patients achieved enteral autonomy with short-term treatment, which is in line with the findings of STEPS-1 [17]. At 30 months, a similar proportion of patients achieved enteral autonomy as patients in both the STEPS-1 and the STEPS-2 studies (25%, *n* = 8, TED-C14-004; 33%, *n* = 30, STEPS-2). Similar to STEPS-3, the 2 patients who achieved enteral autonomy during TED-C14-004 sustained it throughout the extension study SHP633-307 [20]. The similarity in findings between these and the STEPS short- and long-term studies supports the overall efficacy of teduglutide in patients with SBS-IF and demonstrates its equivalent efficacy in Japanese patients compared with European and North American patients. Differences have previously been reported in how long it takes adult patients to respond to teduglutide and whether short-term or longer term treatment is required [19]. The studies discussed here support the notion that longer term treatment of 2 or more years can help more patients with SBS-IF experience clinically meaningful reductions in PS support and possibly achieve enteral autonomy.

The PK data overall were consistent with expected values for teduglutide [13, 31]. These data showed that teduglutide was absorbed quickly and did not accumulate following multiple doses. The inability to detect teduglutide in three patients (TED-C14-004) at the time of PK sampling was attributed to insufficient training in drug administration that resulted in dosing errors. SHP633-306 and SHP633-307 demonstrated that detailed user training in teduglutide administration eliminated the anomalies in plasma drug concentrations observed in TED-C14-004. These results show the potential benefits in efficacy of treatment that can come from ensuring patients have suitable training before administering teduglutide on their own. The AE data reported here are also consistent with the underlying disease and known adverse drug reactions that were reported in previous studies in adults with SBS-IF [17, 32], and no new risks specific to Japanese patients were identified with observations of up to 57 months of treatment. Overall, the PK and safety data support the short- and long-term use of teduglutide 0.05 mg/kg SC once daily for Japanese patients with SBS-IF.

Generally, anti-drug antibodies can reduce the efficacy of drugs as well as cause AEs in patients, resulting in such treatments not being a viable option for patients [33, 34]. However, the development of anti-teduglutide antibodies was not associated with loss of efficacy with short- or long-term treatment, nor was it associated with AEs of hypersensitivity or changes to the PK of teduglutide, which is in line with previous findings [13, 17, 19, 20]. The majority of anti-teduglutide antibodies were detected after 24 weeks of treatment, which is consistent with the findings from the STEPS-1 and STEPS-2 studies [17, 19]. Interestingly, the proportion of Japanese patients with anti-teduglutide antibodies and the titer of those anti-teduglutide antibodies were typically undetectable after 12–24 months of treatment. This is inconsistent with STEPS-1 and STEPS-2, wherein the majority of patients who tested positive for anti-teduglutide antibodies remained positive for those antibodies at ≥ 30 months of treatment [17, 19]. The cause of this discrepancy is unclear, but it is suggested that with long-term treatment, patients may develop immune tolerance to the novel epitope created by the single amino acid substitution that distinguishes teduglutide from native GLP-2 [35].

Limitations associated with these current studies include the small population size and the homogeneity of the population disease etiology, which are a result of the rarity of the condition of focus. The studies lacked a comparator arm, so the possibility cannot be ruled out that reductions in PS might be affected partially by spontaneous intestinal adaptation. However, a period of PS stabilization was required before enrollment, and patients in our studies had been diagnosed with SBS-IF for many years (over 5 years on average) prior to enrollment, well past the expected period of spontaneous adaptation after intestinal resection [36–38]. The increase in urine output, even in the setting of a reduced PS volume, and rapid reductions in PS volumes after initiating teduglutide treatment also indicate that the observed improvements were due to teduglutide treatment.

## Conclusion

In adult Japanese patients with SBS-IF, treatment with teduglutide 0.05 mg/kg SC once daily elicited clinically meaningful reductions in PS requirements. Teduglutide was quickly absorbed and did not accumulate following multiple doses. The effects of teduglutide continued during long-term treatment. No new safety concerns were identified within these short- and long-term studies. These three studies demonstrate that adult Japanese patients with SBS-IF benefit from short- and long-term treatment with teduglutide in a similar manner to patients from North America and Europe. Anti-teduglutide antibody titers, if developed, generally declined to undetectable levels with long-term treatment. These results constitute the longest evaluation of teduglutide treatment within clinical trials reported to date.

## Supplementary Information

Below is the link to the electronic supplementary material.

Supplementary file1 Online Resource 1. Mean changes in the PS volume from baseline up to 24 weeks (a) and beyond 24 weeks (b) of treatment with teduglutide. The week 24 time point for SHP633-306 differs from the month 6 time point for SHP633-307 (SHP633-306 patients) due to differences in the number of patients participating in each study. PS, parenteral support; SE, standard error (PDF 391 KB)

Supplementary file2 Online Resource 2. Mean concentration of plasma citrulline from baseline up to 57 months. SE, standard error (PDF 398 KB)

Supplementary file3 (PDF 182 KB)

## Data Availability

The datasets generated are not publicly available due to the limited number of study participants, such that there is a reasonable likelihood of re-identification of individual patients.
