# Supplementary figures and images for: Efficacy, safety, and pharmacokinetics of teduglutide in adult Japanese patients with short bowel syndrome and intestinal failure: two phase III studies with an extension
Source: Surg Today. 2022 Oct 6;53(3):347–59. doi: 10.1007/s00595-022-02587-4 (PMC9950205; doi:10.1007/s00595-022-02587-4)

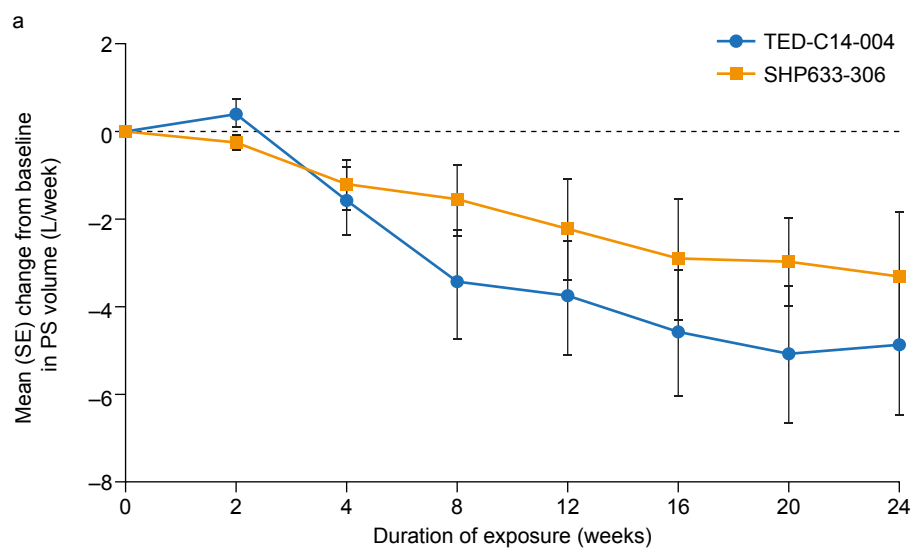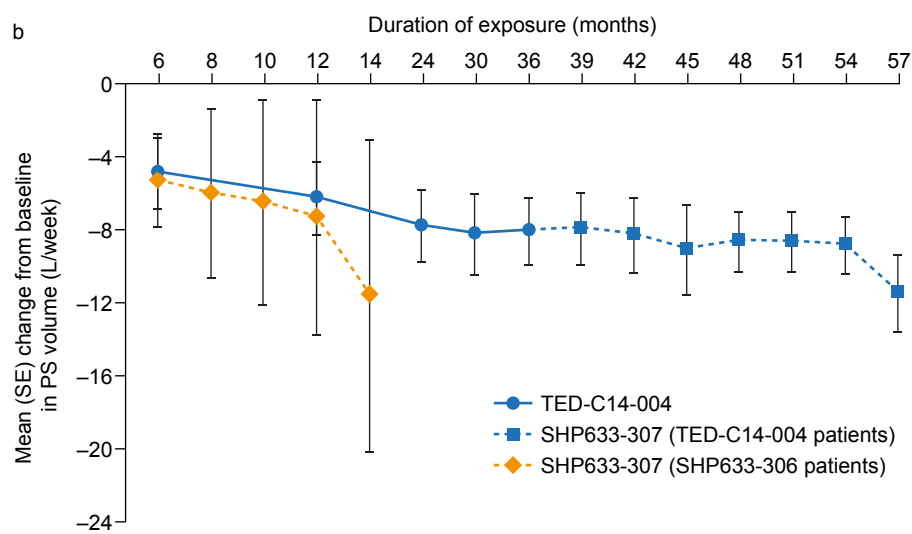

Supplement: Supplementary file 1 — Supplementary file1 Online Resource 1. Mean changes in the PS volume from baseline up to 24 weeks (a) and beyond 24 weeks (b) of treatment with teduglutide. The week 24 time point for SHP633-306 differs from the month 6 time point for SHP633-307 (SHP633-306 patients) due to differences in the number of patients participating in each study. PS, parenteral support; SE, standard error (PDF 391 KB) [file 595_2022_2587_MOESM1_ESM.pdf]

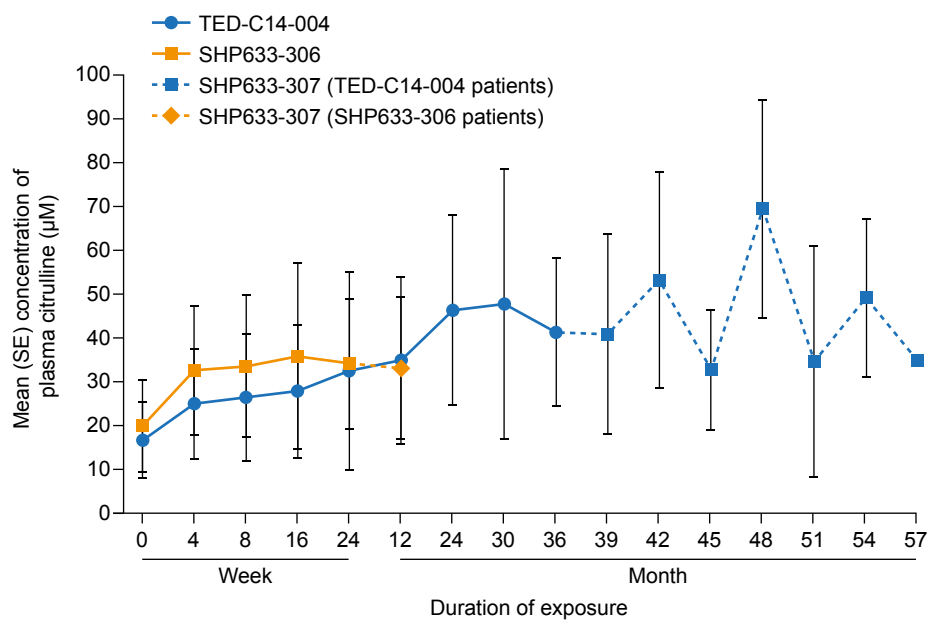

Supplement: Supplementary file 2 — Supplementary file2 Online Resource 2. Mean concentration of plasma citrulline from baseline up to 57 months. SE, standard error (PDF 398 KB) [file 595_2022_2587_MOESM2_ESM.pdf]
