# Supplementary material for: Efficacy, safety, and pharmacokinetics of teduglutide in adult Japanese patients with short bowel syndrome and intestinal failure: two phase III studies with an extension
Source: Surg Today. 2022 Oct 6;53(3):347–59. doi: 10.1007/s00595-022-02587-4 (PMC9950205; doi:10.1007/s00595-022-02587-4)
Supplement: Supplementary file 3 — Supplementary file3 (PDF 182 KB) [file 595_2022_2587_MOESM3_ESM.pdf]

System organ class and preferred term	SHP633-306 and first 24 weeks of TED-C14-004		SHP633-307 and stage 3 – 4 of TED-C14-004 (treatment beyond 24 weeks)		Total	
	Patients n (%)	Events n	Patients n (%)	Events n	Patients n (%)	Events n
**Any TEAE**	17 (94.4)	121	11 (73.3)	199	18 (100.0)	320
**Blood and lymphatic system disorders**	3 (16.7)	4	0	0	3 (16.7)	4
Anemia	2 (11.1)	2	0	0	2 (11.1)	2
**Eye disorder**	2 (11.1)	2	1 (6.7)	5	3 (16.7)	7
**Gastrointestinal disorders**	12 (66.7)	27	8 (53.3)	33	15 (83.3)	60
Abdominal distension	4 (22.2)	4	2 (13.3)	2	6 (33.3)	6
Abdominal pain	5 (27.8)	5	1 (6.7)	1	5 (27.8)	6
Diarrhea	1 (5.6)	1	3 (20.0)	4	4 (22.2)	5
Enteritis	1 (5.6)	1	1 (6.7)	1	2 (11.1)	2
Enterocolitis	1 (5.6)	2	2 (13.3)	2	3 (16.7)	4
Nausea	1 (5.6)	1	3 (20.0)	7	4 (22.2)	8

Stomatitis	1 (5.6)	1	3 (20.0)	3	4 (22.2)	4
Vomiting	2 (11.1)	2	1 (6.7)	1	3 (16.7)	3
**General disorders and administration site conditions**	9 (50.0)	21	5 (33.3)	16	10 (55.6)	37
Catheter site pain	1 (5.6)	1	2 (13.3)	4	2 (11.1)	5
Device occlusion	2 (11.1)	3	2 (13.3)	3	3 (16.7)	6
Injection site reaction	4 (22.2)	4	0	0	4 (22.2)	4
Pyrexia	6 (33.3)	6	4 (26.7)	5	8 (44.4)	11
**Hepatobiliary disorders**	2 (11.1)	3	2 (13.3)	2	4 (22.2)	5
**Immune system disorders**	0	0	2 (13.3)	2	2 (11.1)	2
Hypersensitivity	0	0	2 (13.3)	2	2 (11.1)	2
**Infections and infestations**	8 (44.4)	19	10 (66.7)	62	12 (66.7)	81
Anal abscess	0	0	3 (20.0)	3	3 (16.7)	3
Catheter site infection	1 (5.6)	1	1 (6.7)	1	2 (11.1)	2
Device related infection	6 (33.3)	9	7 (46.7)	24	10 (55.6)	33
Influenza	0	0	5 (33.3)	18	7 (38.9)	21
Nasopharyngitis	3 (16.7)	3	5 (33.3)	18	7 (38.9)	21

Oral herpes	1 (5.6)	1	1 (6.7)	1	2 (11.1)	2
**Injury, poisoning and procedural complications**	4 (22.2)	5	1 (6.7)	1	5 (27.8)	6
**Investigations**	6 (33.3)	9	2 (13.3)	5	7 (38.9)	14
**Metabolism and nutrition disorders**	4 (22.2)	6	5 (33.3)	7	7(38.9)	13
Dehydration	2 (11.1)	2	2 (13.3)	2	2 (11.1)	4
Hypozincemia	1 (5.6)	1	1 (6.7)	1	2 (11.1)	2
**Musculoskeletal and connective tissue disorders**	2 (11.1)	2	6 (40.0)	10	8 (44.4)	12
Arthralgia	0	0	5 (33.3)	5	5 (27.8)	5
Back pain	1 (5.6)	1	1 (6.7)	1	2 (11.1)	2
**Nervous system disorders**	4 (22.2)	7	7 (46.7)	19	8 (44.4)	26
Headache	3 (16.7)	3	5 (33.3)	11	7 (38.9)	14
Somnolence	0	0	3 (20.0)	3	3 (16.7)	3
**Psychiatric disorders**	0	0	3 (20.0)	4	3 (16.7)	4
Insomnia	0	0	2 (13.3)	2	2 (11.1)	2
**Renal and urinary disorders**	2 (11.1)	2	3 (20.0)	4	5 (27.8)	6
Hematuria	1 (5.6)	1	2 (13.3)	2	3 (16.7)	3

**Respiratory, thoracic and mediastinal disorders**	5 (27.8)	5	3 (20.0)	8	7 (38.9)	13
Oropharyngeal pain	1 (5.6)	1	3 (20.0)	3	4 (22.2)	4
Upper respiratory tract inflammation	2 (11.1)	2	1 (6.7)	1	2 (11.1)	3
**Skin and subcutaneous tissue disorders**	4 (22.2)	5	7 (46.7)	13	9 (50.0)	18
Dermatitis contact	0	0	4 (26.7)	4	4 (22.2)	4
Dry skin	1 (5.6)	1	1 (6.7)	1	2 (11.1)	2
Hemorrhage subcutaneous	1 (5.6)	1	2 (13.3)	2	2 (11.1)	3
Pruritus	1 (5.6)	1	1 (6.7)	1	2 (11.1)	2
**Vascular disorders**	2 (11.1)	2	3 (20.0)	4	4 (22.2)	6
Vascular pain	0	0	2 (13.3)	3	2 (11.1)	3

The majority of TEAEs were single events in individual patients.

[a]All device-related infection and device occlusion events were related to the central venous catheter for PS administration, and not to the teduglutide injection device.

PS, parenteral support; *TEAE*, treatment-emergent adverse event
